# Hispidulin Enhances TRAIL-Mediated Apoptosis via CaMKKβ/AMPK/USP51 Axis-Mediated Bim Stabilization

**DOI:** 10.3390/cancers11121960

**Published:** 2019-12-06

**Authors:** Seon Min Woo, Seung Un Seo, Sang Hyun Kim, Ju-Ock Nam, Shin Kim, Jong-Wook Park, Kyoung-jin Min, Taeg Kyu Kwon

**Affiliations:** 1 Department of Immunology, Keimyung University, 1095 Dalgubeoldaero, Dalseo-Gu, Daegu 42601, Korea; woosm724@gmail.com (S.M.W.); sbr2010@hanmail.net (S.U.S.); god98005@dsmc.or.kr (S.K.); j303nih@dsmc.or.kr (J.-W.P.); 2 Deaprtment of Pharmacology, School of Medicine, Kyungpook National University, Daegu 41566, Korea; shkim72@knu.ac.kr; 3 Department of Ecological Environment Conservation, Kyungpook National University, Daegu 41566, Korea; namjo73@gmail.com; 4 New Drug Development Cancer, Deagu-Gyeongbuk Medical Innovation Foundation, 80 Chembok-ro, Dong-gu, Daegu 41061, Korea

**Keywords:** hispidulin, AMPK, USP51, Bim, apoptosis

## Abstract

Hispidulin, a natural compound present in herbs, has anti-cancer effects. Here, we investigated whether hispidulin sensitizes human carcinoma cells to apoptosis induced by TRAIL. Sub-lethal dosages of TRAIL alone and hispidulin alone does not increase apoptosis, but hispidulin increases sensitivity to TRAIL, resulting in induction of apoptosis in hispidulin plus TRAIL-treated cancer cells. In addition, combined treatment with hispidulin and TRAIL also reduced tumor growth and increased apoptosis in xenograft models. However, hispidulin did not alter cell viability in human renal normal mesangial cells and human skin fibroblast. Hispidulin markedly increased the BH3-only proteins Bim at the post-translational levels. Depletion of Bim with siRNA significantly blocked hispidulin plus TRAIL-induced apoptosis. Furthermore, we found that activation of AMPK by hispidulin has a crucial role in Bim proteins stability through up-regulation of USP51 expression. Our findings suggest that USP51-dependent stabilization of Bim by AMPK activation plays a critical role in hispidulin-mediated sensitization of cancer cells to apoptosis induced by TRAIL.

## 1. Introduction

Hispidulin (4′,5,7-trihydroxy-6-methoxyflavone) is a natural compound [1,2], and has multiple functions, including anti-inflammation, anti-fungal, anti-epileptic, anti-hypnotic, and anti-osteoclastogenesis [3,4,5]. In addition, hispidulin has the potential anti-cancer effects. For example, hispidulin induces apoptosis in leukemia and hepatoblastoma cells [6,7], and suppresses angiogenesis, leading to inhibition of tumor growth in xenograft mice models [8]. Anti-cancer effects of hispidulin are mediated by multiple signaling mechanisms, including inhibition of Akt, signal transducer and activator of transcription 3 (STAT3), and aurora kinase [7,9], and activation of AMP-activated protein kinase (AMPK) [10]. Furthermore, hispidulin enhances sensitivity for anti-cancer drugs, such as temozolomide, sunitinib, and tumor necrosis factor-related apoptosis-inducing ligand (TRAIL) [10,11,12]. Sensitization of cancer cells to anti-cancer drug-induced apoptosis is related with down-regulation of apoptosis related proteins (Mcl-1 or Bcl-2) and inhibition of phosphorylation of STAT3 [11,12]. However, the study of mechanisms and the synergistic effects by the combination treatment with sub-lethal concentrations of anti-cancer drugs and hispidulin are still an unclear. 

When TRAIL binds death receptor (DR) 4 or 5, DR recruits Fas-associated death domain (FADD) and caspase-8, which results in formation of death-inducing signal complex (DISC) [13]. In contrast, normal cells highly express decoy receptors, which lack cytoplasmic death domain; thus, TRAIL does not induce apoptosis [14]. However, TRAIL therapy is only limited to TRAIL-sensitive tumor, and a number of cancer cells are resistant to TRAIL [15,16,17,18,19]. To overcome TRAIL resistance, we need more efficient therapeutic strategy, such as combination treatment. Combination treatment might reduce adverse effects, and increase anti-cancer effects [20]. Most of the sensitizers of cancer cells to anti-cancer drugs modulate apoptosis-related proteins or signaling pathways, which are involved in cell survival and proliferation.

Here, we investigate whether hispidulin sensitizes cancer cells to TRAIL, and we identify the molecular mechanisms which involved in hispidulin plus TRAIL-induced apoptosis.

## 2. Results 

### 2.1. Hispidulin Enhances TRAIL-Mediated Apoptosis in Cancer Cells 

**Figure 1:**
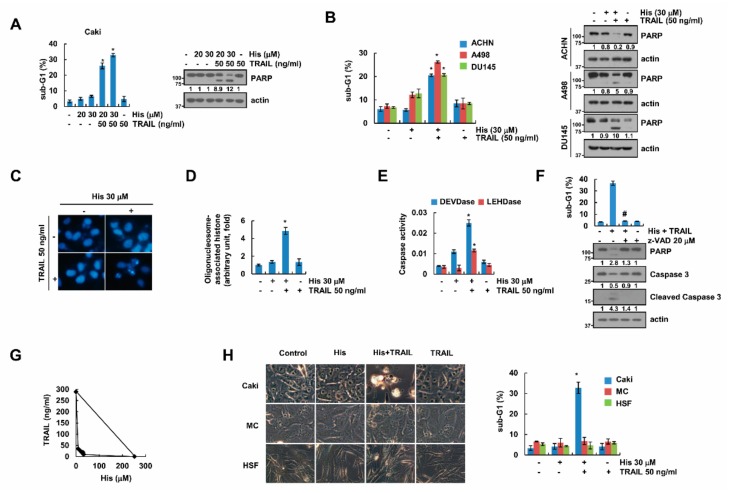
Hispidulin increases the sensitivity of renal carcinoma Caki cells to TRAIL. (**A**,**B**) Human renal carcinoma (Caki, ACHN, and A498) and human prostate carcinoma (DU145) cells were exposed to 50 ng/mL TRAIL and/or the indicated concentrations with hispidulin for 24 h. (**C**–**F**) Caki cells were exposed to 50 ng/mL TRAIL in the presence or absence of the 30 μM hispidulin for 24 h. The condensation and fragmentation of the nuclei were detected by DAPI dye (**C**). The DNA fragmentation and activities of caspase-3 (DEVDase)/9 (LEHDase) are detected using kit as a described in a Material and Methods (**D**,**E**). (**F**) Caki cells were pretreated with 20 μM z-VAD-fmk (z-VAD), and then added with 30 μM hispidulin plus 50 ng/mL TRAIL for 24 h. (**G**) Isoboles were obtained by plotting the combined concentrations of each drug required to produce 50% cell death. The straight line connecting the IC_50_ values obtained for the two agents when applied alone corresponded to the addition of their independent effects. (**H**) Caki, human renal normal mesangial cells (MC) and human skin fibroblast (HSF) cells were treated with 30 μM hispidulin and/or 50 ng/mL TRAIL for 24 h. The cell morphology was detected by interference light microscopy. The percentage of apoptosis was analyzed by measuring the sub-G1 fraction by flow cytometry. The protein levels were determined by Western blotting. The values in A, B, and D–H represent the mean ± SD from three independent experiments. * *p* < 0.05 compared to the control. # *p* < 0.01 compared to the combined treatment with hispidulin and TRAIL.

Hispidulin has anti-cancer effects [6,7] and enhanced anti-cancer drugs-mediated apoptosis [10,11,12]. Therefore, to uncover the novel effect of hispidulin in cancer cells, we investigated whether combination treatment with hispidulin and TRAIL has synergistic effect. First of all, we used sub-lethal concentrations of hispidulin or TRAIL. Combined treatment resulted in induction of sub-G1 population, and we detected cleaved PARP form in combined treated human renal carcinoma Caki cells (Figure 1A) and other human renal carcinoma (ACHN and A498), and human prostate DU145 cancer cells (Figure 1B). Combined treatment with hispidulin and TRAIL presented a variety of apoptosis characteristics, such as nuclear condensation (Figure 1C) and DNA fragmentation (Figure 1D). Additionally, caspase-3 and -9 activities were increased by hispidulin and TRAIL treatment (Figure 1E), and pan-caspase inhibitor (z-VAD) completely inhibited apoptosis and cleavage of PARP and caspase-3 (Figure 1F). Furthermore, we found that hispidulin and TRAIL showed synergistic effects (Figure 1G). Combined treatment with hispidulin and TRAIL induced detachment on plate and cell shrinkage in Caki cells, whereas normal human renal mesangial cells and normal human skin fibroblasts had no response to hispidulin plus TRAIL treatment (Figure 1H). Therefore, these data showed that hispidulin has a synergistic anti-cancer effect with TRAIL on cancer cells. 

### 2.2. Co-Treatment with Hispidulin and TRAIL Reduces Tumor Volume In Vivo

**Figure 2:**
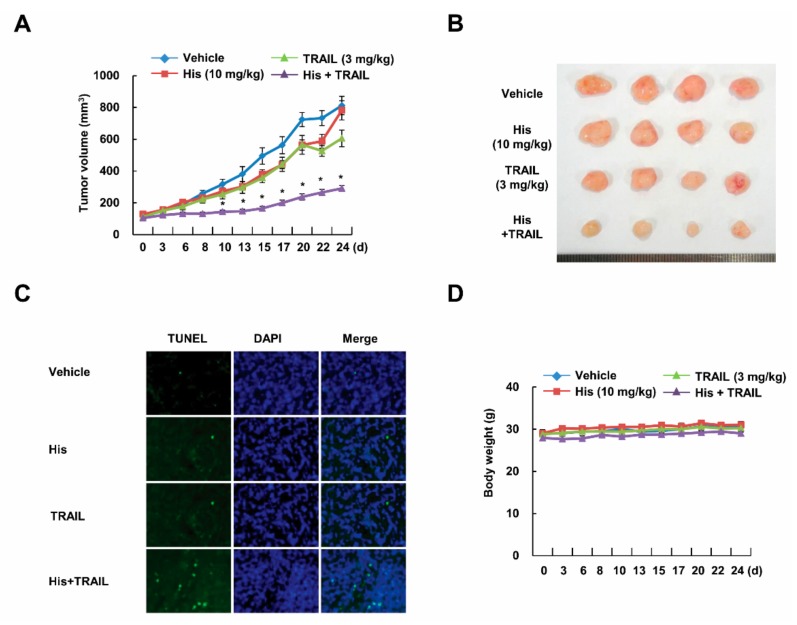
Co-treatment with hispidulin and TRAIL reduces tumor growth in vivo. Caki cells were injected in the flank of nude mice, and then mice were treated three times a week with vehicle, hispidulin (10 mg/kg; intraperitoneal (i.p.)), GST-TRAIL (3 mg/kg, i.p.), or hispidulin plus TRAIL for 21 days. (**A**) The tumor volumes were measured; (**B**) representative tumors are shown; (**C**) representative images of TUNEL assay; (**D**) body weight changes during the experiment. Number of animals per group = 7. Data are means ± SE (n = 7). * *p* < 0.05 compared to vehicle.

To elucidate the anti-tumor effect of hispidulin in vivo, we employed xenograft model. Although single treatment with hispidulin and TRAIL slightly reduced tumor size, combined treatment with hispidulin and TRAIL markedly reduced tumor growth and mass (Figure 2A,B). Consistently, combined treatment increased cell death without weight change (Figure 2C,D). Our data indicate anti-cancer effect of co-treatment with hispidulin and TRAIL in vivo.

### 2.3. Hispidulin Induces Loss of Mitochondrial Membrane Potential 

**Figure 3:**
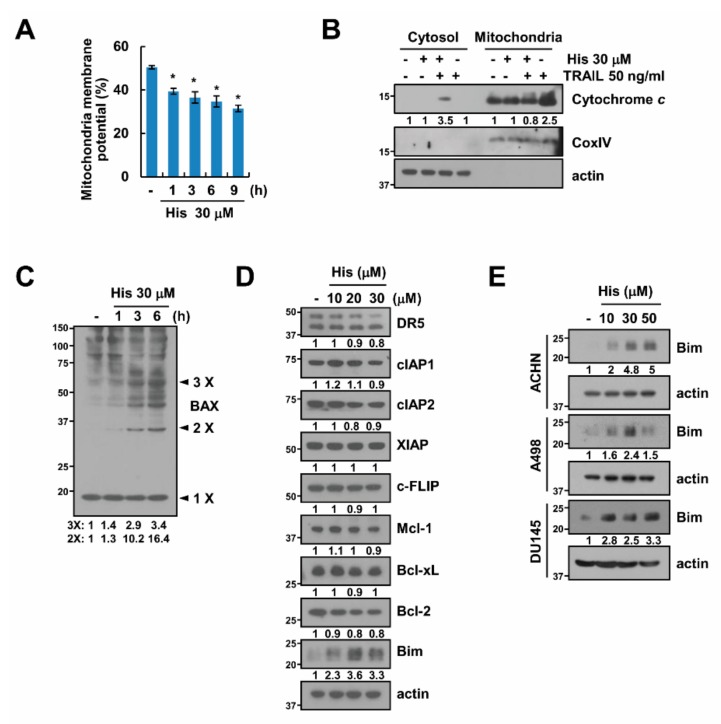
The effect of hispidulin on the mitochondrial membrane potential (MMP). (**A**) Human renal carcinoma Caki cells were exposed to 30 μM hispidulin for the indicated time periods. MMP was detected by rhodamine123 fluorescent dye; (**B**) Caki cells were exposed to 30 μM hispidulin and/or 50 ng/mL TRAIL for 24 h. Cytochrome *c* release is analyzed in cytoplasmic fractions. Cytochrome *c* oxidase subunit IV (COX IV) used as a marker of mitochondria fraction; (**C**) Caki cells were exposed to 30 μM hispidulin for the indicated time periods, and then, Bax monomers and oligomers were detected by Western blotting. (**D**,**E**) Caki, ACHN, A498, and DU145 cells were treated with 10–30 μM hispidulin for 24 h. The expression levels of protein were determined by Western blotting. Data in **A** are presented as the mean ± SD from three independent experiments. * *p* < 0.05 compared to the control.

Next, we want to identify the potential mechanism that is associated with a synergistic anti-tumor effect of hispidulin and TRAIL. First, since release of cytochrome *c* into cytoplasm is a critical to induce apoptosis via the loss of mitochondria membrane potential (MMP) [21], we investigated whether hispidulin induces loss of MMP. Hispidulin induced MMP loss within 1 h (Figure 3A), and cytochrome *c* release was also detected in hispidulin and TRAIL-treated cells (Figure 3B). Previous studies reported cytochrome *c* is released from mitochondria via Bax activation [22] We also detected Bax activation via oligomerization in hispidulin-treated cells (Figure 3C). Moreover, hispidulin significantly induced Bim expression in a dose-dependent manner, but other apoptosis-related proteins were not changed (Figure 3D). Similar results were obtained in hispidulin-treated other cancer cells and in vivo samples (Figure 3E and Supplementary Figure S1). Our data suggest that hispidulin induces MMP loss via Bax activation and induces upregulation of Bim expression. 

### 2.4. Stabilization of Bim Is Involved in Combined Treatment-Induced Apoptosis

**Figure 4:**
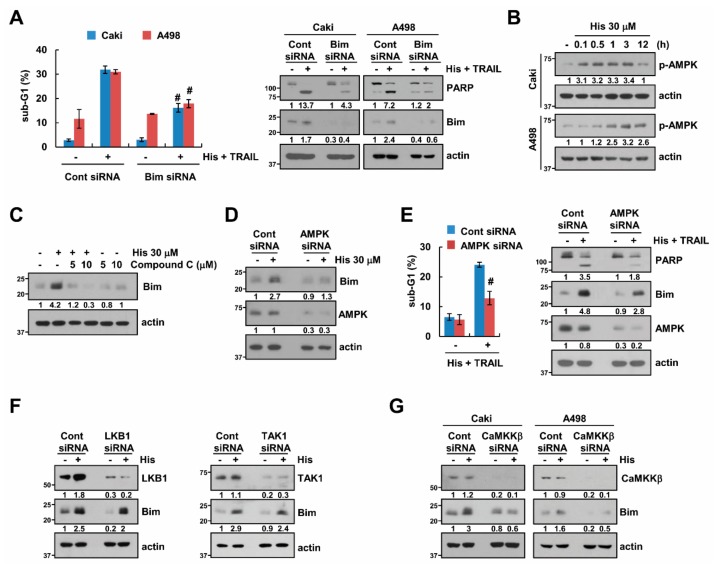
Bim is required for hispidulin-induced sensitization of cancer cells to TRAIL-induced apoptosis. (**A**) Human renal carcinoma Caki and A498 cells were transiently transfected with Bim siRNA or control siRNA. After transfection, cells were exposed to 30 μM hispidulin and 50 ng/mL TRAIL for 24 h; (**B**) Renal carcinoma (Caki and A498) cells were exposed to 30 μM hispidulin for the indicated time periods; (**C**) Caki cells were pretreated with compound C for 30 min, and then exposed to 30 μM hispidulin for 24 h; (**D**,**E**) Caki cells were transiently transfected control (Cont) or AMPK siRNA. After transfection, cells were exposed to 30 μM hispidulin (**D**) or 30 μM hispidulin plus 50 ng/mL TRAIL for the 24 h (**E**). (**F**,**G**) Caki and/or A498 cells were transiently transfected control (Cont. siRNA), LKB1, TAK1 siRNA or CaMKKβ siRNA. After transfection, cells were exposed to 30 μM hispidulin for the 24 h. The level of apoptosis was analyzed by measuring the sub-G1 fraction using flow cytometry. The protein levels were determined by Western blotting. The values in panel **A** and **E** represent the mean ± SD from three independent experiments. # *p* < 0.05 compared to the hispidulin plus TRAIL in control siRNA.

Next, a knock-down of Bim by siRNA was performed to investigate whether an increase in the expression of Bim is involved in the synergistic anti-tumor effect of hispidulin and TRAIL. Down-regulation of Bim expression by two independent siRNAs inhibited apoptosis and PARP cleavage in hispidulin plus TRAIL-treated renal carcinoma cells (Caki and A498) (Figure 4A and Supplementary Figure S2). Previous studies reported that AMPK activation is associated with up-regulation of Bim expression [23,24]. We found that hispidulin induced phosphorylation of AMPK in human renal carcinoma Caki and A498 cells (Figure 4B), and AMPK inhibitor (compound C) inhibited hispidulin-mediated Bim expression (Figure 4C). Additionally, knock-down of AMPK by siRNA inhibited hispidulin-induced Bim expression, and blocked hispidulin plus TRAIL-induced apoptosis (Figure 4D,E). Liver kinase B1 (LKB1), TGF-beta-activated kinase 1 (TAK1), and Calcium/calmodulin dependent protein kinase kinase β (CaMKKβ) are a major up-stream kinases of AMPK [25]. Therefore, we investigated how hispidulin activates AMPK signaling pathway. As shown in Figure 4F, knock-down of LKB1 and TAK1 expression had no effect on hispidulin-induced Bim expression. However, knock-down of CaMKKβ inhibited hispidulin-induced Bim expression in human renal carcinoma Caki and A498 cells (Figure 4G). Therefore, our data demonstrated that hispidulin increased Bim expression via CaMKKβ-AMPK signaling pathway.

### 2.5. AMPK Increases Bim Protein Stability

**Figure 5:**
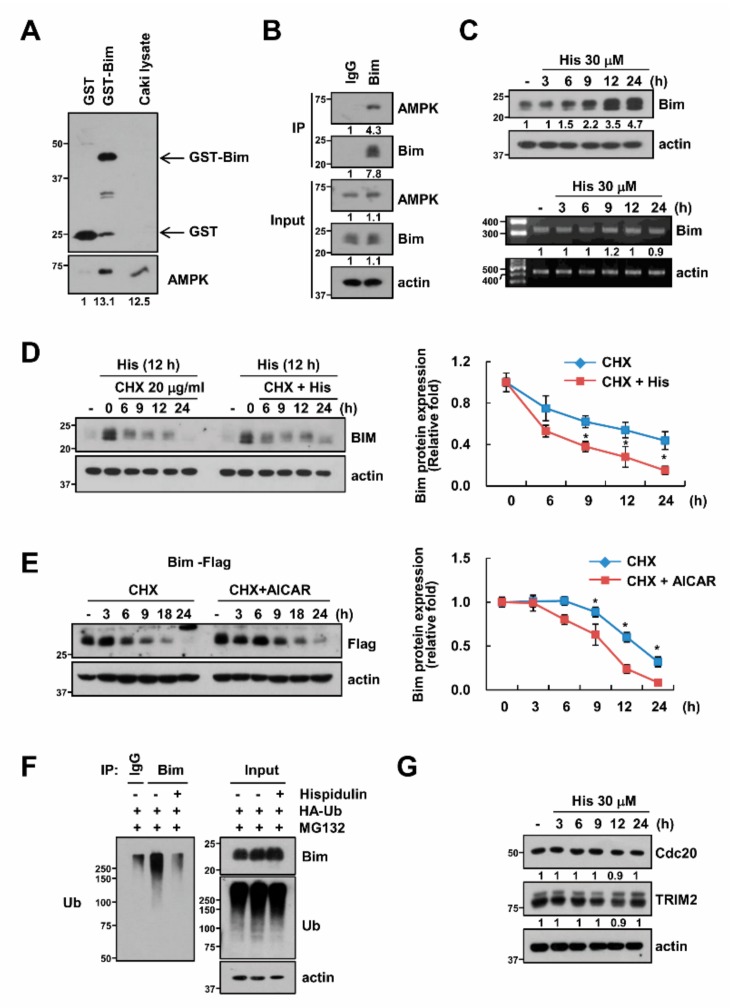
Hispidulin stabilizes Bim via AMPK activation. (**A**) GST and GST-Bim were incubated with whole Caki cell lysates for 6 h; (**B**) The interactions with AMPK and Bim was indicated by immunoprecipitation (IP) assay; (**C**) Caki cells were exposed to 30 μM hispidulin for the indicated time periods. The protein and mRNA levels were determined by Western blotting or RT-PCR, respectively; (**D**) Caki cells were exposed to 20 μg/mL CHX in the presence or absence of 30 μM hispidulin for the indicated time kinetics. The band intensity of Bim was analyzed using ImageJ; (**E**) Caki cells were treated with 20 μg/mL CHX in the presence or absence of 2 mM AICAR for the indicated time kinetics. The band intensity of Bim was analyzed using ImageJ; (**F**) To analyze the ubiquitination of endogenous Bim, Caki cells were transfected with HA-ubiquitin (HA-Ub) and treated with 0.5 μM MG132 and 30 μM hispidulin. Cells were lysed in 1% SDS buffer to disrupt interacting proteins and cell lysates were then diluted to 0.1% SDS, followed by the immunoprecipitation using an anti-Bim. Bim ubiquitination was detected by immunoblot analysis using an HRP-conjugated anti-Ub antibody; (**G**) Caki cells were treated with 30 μM hispidulin for the indicated time periods. The protein levels were determined by Western blotting.

We carried out a GST-full down experiment to investigate the direct relevance of AMPK to the control of Bim expression. As shown in Figure 5A, AMPK was bound to GST-Bim but not to the GST-control. Furthermore, to confirm the interaction between AMPK and Bim, we performed the co-immunoprecipitation of the complex. We found that AMPK was interacted with Bim (Figure 5B). AMPK has been known to modulate the Bim expression in the transcriptional levels [26] or in the post-translational levels [23]. In our conditions, hispidulin did not induce changes in the mRNA expression of Bim (Figure 5C). Therefore, to identify the possibility of post-translational modulation, cells were treated with cycloheximide (CHX; inhibitor of de novo protein synthesis) in the presence or absence of hispidulin. The combined treatment with CHX and hispidulin increased Bim stability (Figure 5D. In addition, Bim stability was also increased by AICAR treatment, an AMPK activator (Figure 5E). Since the protein stability of Bim could be regulated through ubiquitination [27,28], we tested the effect of hispidulin on the ubiquitination of Bim. As shown in Figure 5F, hispidulin markedly decreased polyubiquitination of Bim. Therefore, we examined the change of E3 ligase levels of Bim, such as cdc20 [29] and TRIM2 [30]. However, hispidulin did not alter expression levels of Cdc20 and TRIM2 (Figure 5G). Our data indicate that hispidulin increases Bim protein stability in an AMPK dependent manner. 

### 2.6. USP51 Modulates Hispidulin-Induced Bim Stabilization

**Figure 6:**
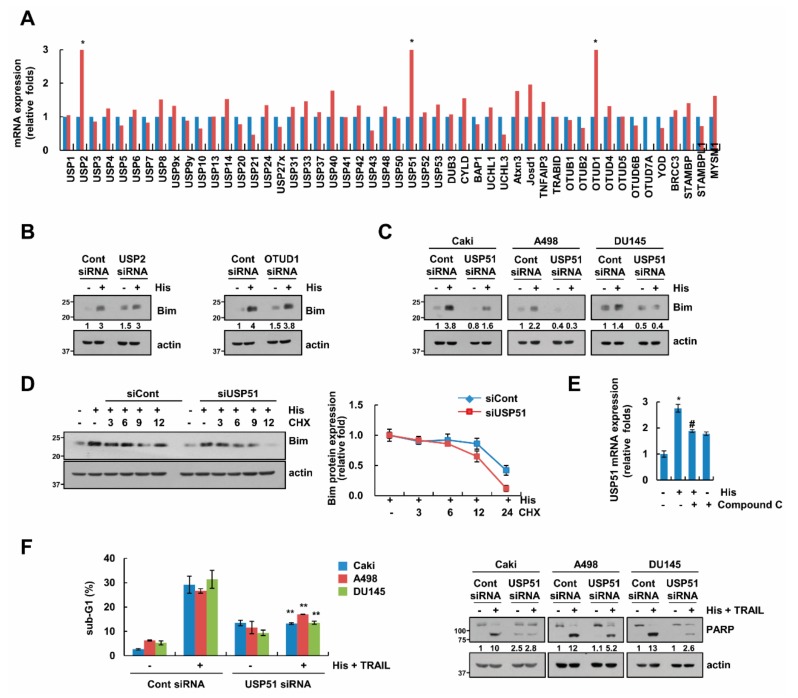
USP51 is involved in hispidulin-induced Bim stabilization. (**A**) Caki cells were exposed to 30 μM hispidulin for 24 h. DUB expression was detected by real time PCR; (**B**,**C**) Caki, A498, and/or DU145 cells were transiently transfected with USP2, OTUD1, USP51, or control siRNA. After transfection, cells were exposed to 30 μM hispidulin for 24 h. The protein levels were determined by Western blotting; (**D**) Caki cells were transiently transfected with USP51 or control siRNA. Overnight after transfection, cells were treated with 20 μg/mL cycloheximide (CHX) and 30 μM hispidulin for the indicated time kinetics. The protein and mRNA levels were determined by Western blotting. The band intensity of Bim was analyzed using ImageJ; (**E**) Caki cells were pretreated with 10 μM compound C for 30 min, and then treated with 30 μM hispidulin for 24 h. USP51 mRNA was detected by real time PCR; (**F**) Caki, A498, and DU145 cells were transiently transfected with USP51 or control siRNA. After transfection, cells were exposed to 30 μM hispidulin plus 50 ng/mL TRAIL for 24 h. The level of apoptosis was analyzed by measuring the sub-G1 fraction by flow cytometry. The protein levels were determined by Western blotting. The values in panel **A**, **D**, **E**, and **F** represent the mean ± SD from three independent experiments. * *p* < 0.05 compared to the control. # *p* < 0.05 compared to the hispidulin. ** *p* < 0.05 compared to the hispidulin plus TRAIL in control siRNA.

To identify the novel mechanism of Bim protein stability, we screened the deubiquitinases (DUBs) expression in hispidulin-treated cells. Among the DUBs, mRNA expression levels of ubiquitin specific protease 2 (USP2), OTU deubiquitinase 1 (OTUD1), and USP51 were increased by hispidulin treatment (Figure 6A). Therefore, we investigated whether these DUBs are involved in hispidulin-induced Bim stabilization. Knock down of USP51 only blocked hispidulin-induced Bim stabilization (Figure 6B,C), and the knock down of USP51 decreased Bim stability in the presence of hispidulin and CHX (Figure 6D). In addition, to confirm the relevance of USP51 and AMPK, cells were treated with hispidulin in the presence of absence of compound C. Inhibition of AMPK inhibited hispidulin-induced USP51 expression (Figure 6E). To verify the involvement of USP51 upregulation in hispidulin-induced TRAIL sensitization, we examined the apoptosis using USP51 siRNA. Inhibition of USP51 partially blocked apoptosis by hispidulin plus TRAIL in Caki, A498 and DU145 cells (Figure 6F). Therefore, our data suggested that hispidulin increased Bim protein stability via AMPK-induced USP51 expression. 

## 3. Discussion

**Figure 7:**
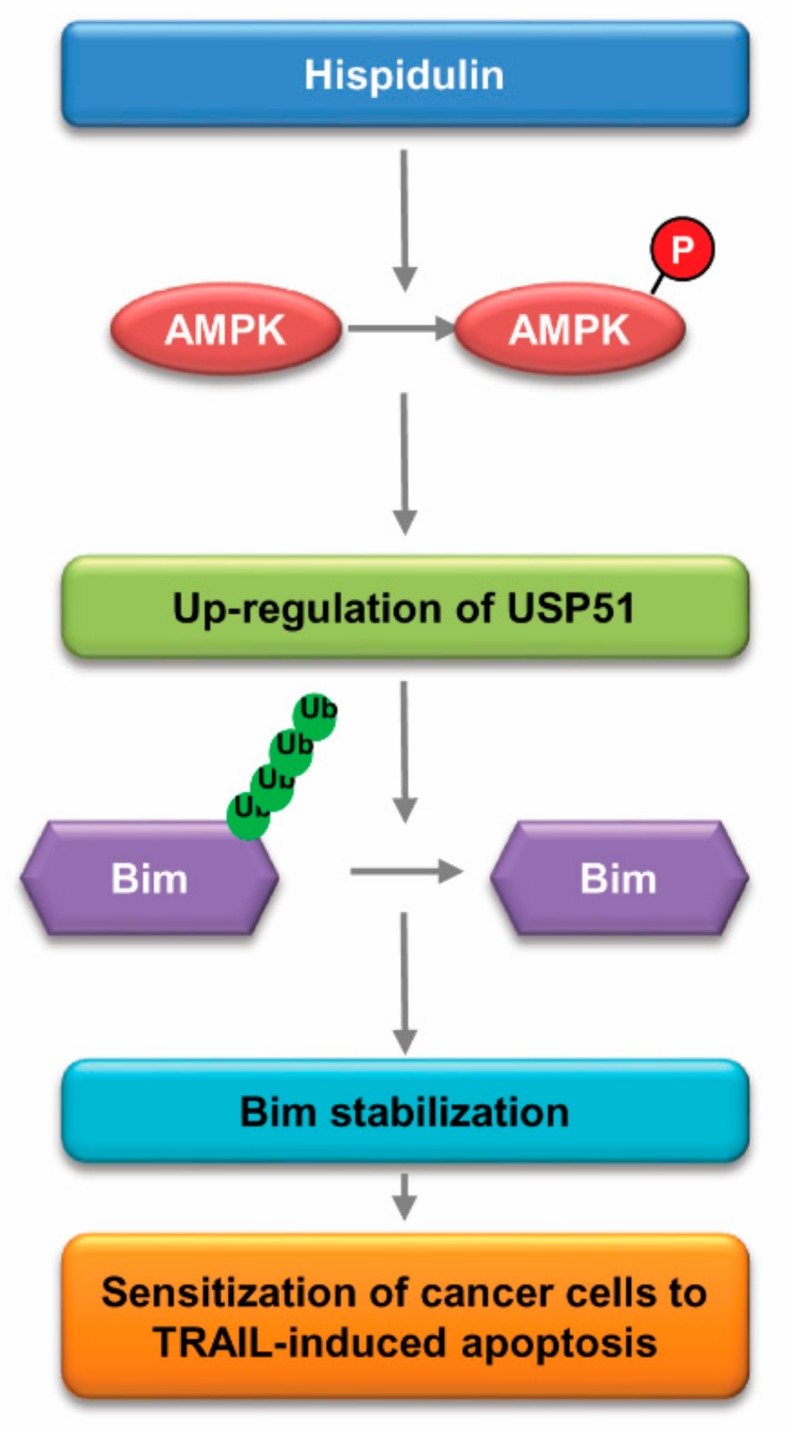
Scheme indicating the mechanism to sensitize cancer cells to TRAIL-mediated apoptosis induced by hispidulin.

Here, we demonstrated that combined treatment with hispidulin and TRAIL has synergistic anti-cancer effects in cancer cells and in *in vivo* xenograft models. We found that stabilization of Bim by hispidulin is a critical factor for combined treatment-induced apoptosis, and AMPK activation is related with Bim stabilization. AMPK increased Bim protein stability via up-regulation of USP51 expression in hispidulin-treated cells (Figure 7). Therefore, we suggested that hispidulin could enhance TRAIL-mediated apoptosis via CaMKKβ/AMPK/USP51axis-mediated Bim stabilization.

Unlike other members of DR-mediated apoptosis inducers, TRAIL induces selectively apoptosis in cancer cells. This merit develops TRAIL receptor agonistic antibody or recombinant TRAIL (Dulanermin) as a cancer treatment in the clinical studies [31,32,33]. However, although anticancer activities of some cases are demonstrated, there are no detected statistically significance in most cases of clinical trials. Lemke et al. discussed about the three causes of failure in TRAIL clinical trials [34]. First, to reduce the hepatotoxicity shown in previous attempts to use TNF-, CD95L, and CD95 agonistic antibodies, the TRAIL receptor agonist with low agonistic activity was used. The second is to try monotherapy even though there are cells that have TRAIL resistance. Third, patients participated in TRAIL receptor agonist clinical trials were not selected on the basis of valid biomarkers [34]. To compensate for these problems, it should be developed the highly agonistic TRAIL receptor agonist or stable recombinant TRAIL. In addition, the discovery of novel TRAIL sensitizers improves anti-cancer activity and reduces the adverse effects. 

AMP-activated protein kinase (AMPK) is serine/threonine protein kinase, and is composed of a catalytic subunit α and two regulatory subunits, β and γ [35]. For kinase activation, the phosphorylation of AMPKα subunit is necessary, and this phosphorylation is mainly regulated by three upstream kinases, LKB1, TAK1, and CaMKKβ [25]. In our study, down-regulation of CaMKKβ inhibited hispidulin-induced Bim stabilization (Figure 4G). However, LKB1 and TAK1 were not associated with hispidulin-induced Bim stabilization (Figure 4F). Therefore, at least, hispidulin activates AMPK signaling pathways via CaMKKβ activation in human renal carcinoma Caki cells. The functions of AMPK are controversial in cancer cells, depending on cell types, stimulators, and duration of activation. Multiple anti-cancer drugs, including simvastatin [36], phenformin [37], vincristine [38], and doxorubicin [39], induced apoptosis via AMPK activation [36,37,38,39]. However, activation of AMPK by parthenolide [29] and bromodomain and extraterminal domain (BET) inhibitors (JQ1) [40] inhibits induction of apoptosis, and berberine-induced AMPK inhibition induced apoptosis [41]. Hispidulin increased pro-apoptotic Bim stabilization via AMPK activation at the post-translational levels (Figure 4D and Figure 5C). Furthermore, down-regulation of AMPK by siRNA inhibited apoptosis in hispidulin plus TRAIL-treated cells. Therefore, hispidulin-induced AMPK activation is involved in apoptosis in human renal carcinoma Caki cells. Yang et al. also reported that hispidulin enhanced TRAIL-mediated apoptosis via AMPK activation in human ovarian cancer cells [12]. Hispidulin induced down-regulation of Mcl-1, Bcl-2, and Bcl-xL expression via mTOR inhibition by AMPK activation [12]. Although hispidulin activates AMPK signaling pathway, the effect of hispidulin on apoptosis-related proteins is different, depending on cell types.

Bim is the only BH3 protein controlling cell death by binding anti-apoptotic proteins and activation of Bax and Bak. Down-regulation of Bim protein expression induces an anti-cancer drug resistance and inhibits cell death in cancers [42,43,44]. Therefore, up-regulation of Bim protein expression is targeted to induce cancer cell death. The modulation of Bim expression is regulated at the transcriptional levels and at the post-translational levels. Here, we identified that hispidulin increased Bim expression via protein stabilization (Figure 5D). Degradation of Bim is mainly mediated by proteasome via ubiquitination [27,28]. Several papers reported about E3 ligase or DUBs of Bim. For examples, cdc20 [29] and tripartite motif-containing protein 2 (TRIM2) [30] increases ubiquitination of Bim, resulted in degradation. However, in our study, hispidulin did not induce significant change of both cdc20 and TRIM2 expression (Figure 5G). In addition, transducin repeats-containing proteins (β-TrCP) is also E3 ligase of Bim, and the interaction between β-TrCP and Bim is dependent of ERK-mediated phosphorylation of Bim [45]. Hispidulin-induced Bim stabilization was not changed by ERK inhibitor in Caki cells (data not shown). Therefore, we thought it was unlikely that β-TrCP would control the expression of Bim in hispidulin-treated cells. Therefore, we found a relatively small number of DUBs compared to ubiquitinases in order to identify the mechanism in which hispidulin can control the stabilization of the Bim. As shown in Figure 6A, three DUBs, namely USP2, OTUD1, and USP51, were markedly increased by hispidulin treatment. However, only knockdown of USP51 blocked up-regulation of Bim stabilization by hispidulin (Figure 6C). Weber et al. reported that USP27x induced stabilization of Bim [27]. USP27x, similar to β-TrCP, is known to recognize and bind to Bim in ERK-mediated phosphorylation dependent manner [27]. As mentioned above, the relevance of these USP9x cannot be completely ruled out because the increase in the stabilization of Bim by hispidulin is irrelevant to ERK, but there will be little control of the Bim stabilization through USP9X. We identified a new DUB that can increase the stability of Bim in this study. However, further molecular mechanism study of USP51-mediated Bim stabilization is required. 

Collectively, we suggest that USP51-dependent stabilization of Bim by AMPK activation has an important role in hispidulin-mediated sensitization of cancer cells to TRAIL-induced apoptosis. 

## 4. Materials and Methods

### 4.1. Cell Cultures and Materials

We obtained the normal human renal mesangial cells from Lonza (CC-2559, Basel, Switzerland), and other cells lines (Caki (HTB-46), ACHN (CRL-1611), A498 (HTB-44), and DU145 (HTB-81)) purchased from ATCC (Manassas, VA, USA). The normal human skin fibroblasts cells were purchased form Korea Cell Line Bank (Seoul, Korea). Cell lines are cultured with dulbecco’s modified eagle medium (DMEM) containing 10% fetal bovine serum (FBS) and 1% 100 μg/mL gentamicin. PCR primers were purchased from Macrogen (Seoul, Korea). GST-TRAIL cDNA plasmid was a gift from Dr. Kim YS (Ajou University, Suwon, Korea). Recombinant human TRAIL and z-VAD-fmk was purchased from R&D system (Minneapolis, MN, USA). Hispidulin was purchased from Santa Cruz Biotechnology (Santa Cruz, CA, USA). Compound C was purchased from Calbiochem (Darmstadt, Germany). Anti-Mcl-1, anti-Bcl-xL, anti-Bcl-2, anti-cIAP2, anti-Cox IV, anti-ubiquitin, anti-LKB1, anti-TAK1, anti-CaMKKβ, and anti-AMPK antibodies were purchased from Santa Cruz Biotechnology (Santa Cruz, CA, USA). Anti-Bim was purchased from Millipore Corporation (Billerica, MA, USA). Anti-caspase-3 antibody was purchased from ENZO (Ann Arbor, MI, USA). Anti-cytochrome *c*, anti-Bax and anti-XIAP antibodies were purchased from BD Biosciences (San Jose, CA, USA). Anti-PARP, anti-cleaved caspase-3, anti-DR5, anti-cIAP1, and anti-pAMPK antibodies were purchased from Cell Signaling Technology (Beverly, MA, USA). Anti-cdc20 and anti-c-FLIP antibodies were purchased from Abcam, (Cambridge, MA, USA), and Enzo Life Science (Farmingdale, NY, USA), respectively. Anti-TRIM2 and anti-actin antibodies were obtained from Sigma–Aldrich (St. Louis, MO, USA). Other reagents were purchased from Sigma Chemical Co. (St. Louis, MO, USA). 

### 4.2. Flow Cytometry Analysis

For flow cytometry, the cells were harvest and fixed with 70% ethanol at 4 °C for 1 h. Fixed cells were washed with phosphate buffered saline (PBS), and incubated with 50 μg/mL RNase for 30 min at 37 °C [46]. The DNA was stained with 50 μg/mL propidium iodide solution for 30 min at room temperature, and the cellular DNA content was detected with FACS Calibur flow cytometer (BD Biosciences). Apoptotic cells contain fragmented DNA, and this appears as sub-G1 peak on the histogram. Therefore, we represented apoptosis as a sub-G1 population.

### 4.3. Western Blot Analysis

Cells were lysed on ice in 50 μL of RIPA (radioimmunoprecipitation assay) lysis buffer, and were centrifuged at 10,000× *g* for 15 min at 4 °C. Protein extracts were separated by sodium dodecyl sulfate polyacrylamide gel electrophoresis (SDS–PAGE) gels by electrophoresis and transferred to Immobilon-P membrane. Membranes were treated at 4 °C for 1 h in 5% non-fat milk-1X tris-buffered saline, 0.1% Tween® 20 (TBST), and then incubated overnight with primary antibodies. After washing, membranes were treated with secondary antibodies for 1 h. Specific proteins were detected using an enhanced chemiluminescence (ECL) Western blot kit according to the manufacturer’s instructions. Full blots of cropped images are included in Supplementary Figures S3–S8.

### 4.4. Determination of Synergy

The possible synergistic effect of hispidulin and TRAIL was analyzed using the isobologram method. Cells were treated with different concentrations of hispidulin and TRAIL alone or in combination. After 24 h, XTT (sodium 3′-[1-[(phenylamino)-carbony]-3,4-tetrazolium]-bis(4-methoxy-6-nitro)benzene-sulfonic acid hydrate) assay was employed to measure the cell viability using WelCount Cell Viability Assay Kit (WelGENE, Daegu, Korea). Relative survival was assessed, and the concentration effect curves were used to determine the IC50 (the half-maximal inhibitory concentration) values for each drug alone and in combination with a fixed concentration of the second agent [47].

### 4.5. 4′,6′-Diamidino-2-Phenylindole Staining (DAPI) for Nuclei Condensation and Cell Death Assessment by DNA Fragmentation Assay

To identify apoptosis, nuclear morphology was stained with 4′,6′-diamidino-2-phenylindole (DAPI) (Roche, Mannheim, Germany), and detected by fluorescence microscopy. To detect fragmented DNA, we used the cell death detection ELISA plus kit (Boehringer Mannheim, Indianapolis, IN, USA). Cells were lysed, and then centrifuged at 200× *g* for 10 min. Lysates were used for detection of nucleosomes in cytoplasmic fractions by spectrophotometry. 

### 4.6. Asp-Glu-Val-Asp-Ase (DEVDase) Activity Assay

Cells were lysed in 100 μL of reaction buffer, and then 20 μg of cell lysates were incubated with 5 μM caspase substrate [Asp-Glu-Val-Asp-chromophore-p-nitroanilide (DVAD-pNA)]. The absorbance at 405 nm was measured by spectrophotometer.

### 4.7. In Vivo Xenograft Model

Male BALB (bagg and albino)/c-nude mice were purchased from the Central Lab Animal Inc. (Seoul, Korea), and mice were maintained in a pathogen-free conditioned room. The IRB Keimyung University Ethics Committee (KM-2013–82) approved our study protocol. Caki cells (2 × 10^6^) were subcutaneously injected on each flank. After Caki xenograft had grown after 2 weeks, 28 mice were randomly divided into vehicle alone, hispidulin alone, GST-TRAIL alone, and hispidulin plus GST-TRAIL. The mice received an intraperitoneal (i.p.) injection of 10 mg/kg (in PBS) hispidulin and 3 mg/kg (in PBS) GST-TRAIL three times a week for 3 weeks. The tumor size (length × width^2^)/2 was measured every time using a Vernier’s caliper (Mytutoyo Co., Tokyo, Japan). The animals were sacrificed by cervical dislocation. Apoptosis was detected ApopTag Fluorescein in situ Apoptosis Detection Kit (Millipore). 

### 4.8. Determination of the Mitochondrial Membrane Potential 

After treatment, cells were incubated with 5 μM rhodamine 123 (Molecular Probes Inc., Eugene, OR, USA) for 5 min in the dark at 37 °C. The mitochondrial membrane potential was analyzed by FACS Calibur flow cytometer (BD Biosciences). 

### 4.9. Preparation of Cytosolic and Mitochondrial Fractions

The cells were lysed in 80 μL ice-cold lysis buffer [250 mM sucrose, 1 mM ethylenediaminetetraacetic acid (EDTA), 20 mM Tris–HCl (pH 7.2), 1 mM DTT, 10 mM KCl, 1.5 mM MgCl_2_], and were centrifuged at 12,000× *g* at 4 °C for 10 min to obtain the supernatants (cytosolic extracts free of mitochondria) and the pellets (fraction that contains mitochondria). 

### 4.10. Assay for Bax Activation and Oligomerization

For Bax oligomerization, the cells were incubated with conjugation buffer containing 10 mM EDTA, and 0.2 mM Bismaleimide (Thermo Scientific, Hudson, NH, USA) was added at room temperature for 1 h. Lysates were extracted by lysis buffer for Western blot analysis.

### 4.11. GST Protein Purification and GST Pull-Down Assay

Recombinant GST and GST-Bim fusion proteins were generated by expressing the appropriate pGEX-4T1 constructs in the BL21 strain of *Escherichia coli*. The expression of the recombinant protein was induced with isopropyl β-d-1-thiogalactopyranoside (IPTG) to a final concentration of 0.4 mM (1 L of culture) at 28 °C for 6 h. After induction, the cells were harvested and sonicated in lysis buffer (PBST; PBS, 1% triton X-100), and then, fusion protein were purified using Glutathione Sepharose 4B beads at 4 °C for 2 h with rotation. The purified proteins were incubated with the 1 mg of Caki cell lysates for 2 h at 4 °C with rotation, and then beads washed four times. Bound AMPK was detected through western blotting.

### 4.12. Ubiquitination Assay

This assay was described using the tagged-ubiq uitin plasmid and pretreatment of MG132 [48]. Briefly, cells were harvested, washed with PBS containing 10 mM N-ethylmaleimide (NEM) (EMD Millipore, Darmstadt, Germany), resuspended in 90 μL PBS/NEM containing 1% SDS, and boiled for 10 min at 95 °C. Lysates were added to lysis buffer involving 1 mM phenylmethylsulfonyl fluoride (PMSF) and 5 mM NEM, dissolved using l mL syringe 3–4 times and centrifuged at 13,000× *g* for 10 min at 4 °C. The supernatants were incubated with primary antibody of target protein for overnight and reacted by adding protein G agarose bead for 2 h. After centrifuging, the supernatants were removed, washed with lysis buffer containing 1 mM PMSF and 5 mM NEM two times and boiled using 2X sample buffer for 10 min. Ubiquitinated Bim was detected using HRP-conjugated anti-Ub.

### 4.13. Reverse Transcription Polymerase Chain Reaction and Real Time PCR

Reverse Transcription Polymerase Chain Reaction (RT-PCR) and real time PCR was performed as a described previously [49]. The information of used primer in this study is listed in Table S1. 

### 4.14. Small-Interfering RNAs (siRNAs)

The GFP (control), LKB1, TAK1, CaMKKβ, and AMPK siRNA duplexes were purchased from Santa Cruz Biotechnology (Santa Cruz, CA, USA) and Bim siRNA duplexes is obtained from Bioneer (Dejeon, Korea). The siRNA transfected into Caki cells using Lipofectamine RNAiMAX (Thermo Fisher Scientific, Waltham, MA, USA). 

### 4.15. Statistical Analysis

The data were analyzed using a one-way ANOVA and post-hoc comparisons (Student-Newman-Keuls) using the Statistical Package for Social Sciences 22.0 software (SPSS Inc., Chicago, IL, USA). 

## 5. Conclusions

In conclusion, we suggest that hispidulin enhances TRAIL-mediated apoptosis in cancer cells, and the mechanism is that hispidulin induces Bim stabilization through CaMKKβ/AMPK-mediated USP51 expression. These findings provide new concepts that hispidulin sensitizes cancer cells to TRAIL-induced apoptosis via CaMKKβ/AMPK/USP51-mediated Bim stabilization as a therapeutic strategy for cancer treatment. 

## Supplementary Materials

The following are available online at https://www.mdpi.com/2072-6694/11/12/1960/s1, Figure S1: Effect of hispidulin on Bim expression in tumor tissues; Figure S2: Bim is critical for hispidulin plus TRAIL-induced apoptosis; Figure S3: Uncropped image for Figure 1; Figure S4: Uncropped image for Figure 3; Figure S5: Uncropped image for Figure 4; Figure S6: Uncropped image for Figure 5; Figure S7: Uncropped image for Figure 6; Figure S8: Uncropped image for supplementary Figures S1 and S2; Table S1: The primer sequences.

## Abbreviations

TRAIL	Tumor necrosis factor-related apoptosis-inducing ligand
PARP	poly (ADP-ribose) polymerase
MMP	Mitochondrial membrane potential
FADD	Fas-associated death domain
DR	death receptor
AMPK	5’ AMP-activated protein kinase
LKB1	Liver kinase B1
TAK1	TGF-beta-activated kinase 1
CaMKK	Calcium/calmodulin dependent protein kinase kinase
STAT3	Signal transducer and activator of transcription 3
β-TrCP	β-transducin repeats-containing proteins
USP	Ubiquitin specific peptidase
OTUD1	OTU deubiquitinase 1
