# Supplementary material for: Hispidulin Enhances TRAIL-Mediated Apoptosis via CaMKKβ/AMPK/USP51 Axis-Mediated Bim Stabilization
Source: Cancers (Basel). 2019 Dec 6;11(12):1960. doi: 10.3390/cancers11121960 (PMC6966507; doi:10.3390/cancers11121960)
Supplement: Supplementary file 1 [file cancers-11-01960-s001.zip › Supplementary Table 1.docx]

**Supplementary Table 1. The primer sequences**

| Name | Forward | Reverse |
| --- | --- | --- |
| Bim | CACAAAACCCCAAGTCCTCCTT | TTCAGCCTGCCTCATGGAA |
| USP1 | TCTGTGCCTGCGTTGTTTGA | CGTCCTTTGAAATTGCCGGT |
| USP2 | GTTCGACTCGTCCATACTCCA | GGGTGTAGGCACCATAGCC |
| USP3 | GGAAGCCCGTGCTTTCTTTG | ACCCAAGGGCTTTTGTTGGA |
| USP4 | ACACCTACGAGCAGTTGAGC | GGCGCAGTGCTTGATTTTGA |
| USP5 | TGTCCATCGAGATGCCAGAG | GGAGTCTTCGTCTTCGTTGC |
| USP6 | ACTTGTTGACAATGCACTGACTT | AGCACATAGAGGCAACCACA |
| USP7 | CATGGAGATGGAAGCGGGAG | TCACTCAGTCTGCTGAAGCG |
| USP8 | CGTCACGGAAAGAAGCACTTG | AGGGCACTGTGCACATAACT |
| USP9X | CATGGACCTGGCTCTCAGTG | GGCGCTTCACCAAACAAACT |
| USP9Y | CTGGGCTCAGAGGTGAAACT | GACTAAGTCCACGTCTACCACT |
| USP10 | CTGCCATTCTGTCCCGTCTT | TGCCACACAGAACTGTTCCA |
| USP13 | CAAAAGATCGCCTGATGAACCA | TGATCCAGTTGAAGGCCACC |
| USP14 | CAGCTGTTTGCGTTGACTGG | TTGGCTGAGGGTTCTTCTGG |
| USP20 | TGACTACGGGCAGATTTCGG | GGCGACTTACAGACTAGCCC |
| USP21 | GACCAACTTAGCCCGTTCCA | TACGTCTCAAAGTGGGTGGC |
| USP24 | CATGGAATCGGAGGAGGAGC | ACGGCCTCGTTAATGTCGTT |
| USP27x | CGTCTCCACCACGATAGACC | CTCTGGCCTCGTAAACCTCC |
| USP31 | TCAGCAACACCGAGCTCTTC | TGTACTCCAGGGTCCAGAGG |
| USP33 | TTGCCCACCTTTGACACAGT | GGCCTGCTTTTATGCCACAG |
| USP36 | AGGTCAACTTCGGAGAGGCT | CCAGGATCTTAACGGAGGGC |
| USP40 | CAGAAAGCGTGTGGGATTTGACC | GTGAAGTCCTGCTGGTACAAGC |
| USP41 | TTCAGGGCTCATCAGTGTCA | AGTTAAGGCAGCAGGTCTGTC |
| USP42 | ATGGCCAGGGTGATTGAAAAC | GAAGCACCACGCAGATTGGA |
| USP43 | CTGCCGGAACTCTCTGGATG | CAGACAGGGAGGAGCTGGTA |
| USP48 | GTCCTCTACATCGTGTCTCAGTT | GGGACACAAAAGAGCACTGTTCC |
| USP50 | CAAGTTGGAGTGGTGCCTGA | TCTGAGCCTGTTAACGCTGG |
| USP51 | AAAAGAATGCTTTAGGTGGG | AAGGAATACTCCCTGACTTC |
| USP52 | GGGTAACTAGCCGTTTCCGT | GGTGGGGAGAACGAGAAGTC |
| USP53 | GTACTGTGGCAGCAGTCAGT | ACAGGACGAAGGGTCTTTGC |
| DUB3 | CCTCCCGACGTACTTGTGAT | CATGGACTCCTGATGTGTCG |
| CYLD | TTGGCAACTGGGATGGAAGA | TCCTTTCCTGCGTCACACTC |
| BAP1 | CTACCACGACATCCGCTTCA | CAGGCAGCTGTGACTCTTGA |
| UCHL1 | GGCCCAGCATGAGAACTTCA | TGGCCACTGCGTGAATAAGT |
| UCHL3 | GCATGCGTAGAACGCGA | GCCAATTTCCTATTTCGGGAGA |
| ATXN3 | TCCACGAGAAACAAGAAGGCT | AGCTGATGTGCAATTGAGGA |
| JOSD1 | CACTCCAGGTGGCGGC | GGCCTGGGTGACGGATAAAT |
| TNFAIP3 | GCATACAACTGAAACGGGGC | GGGGTGTGATCTCTCTTGGC |
| TRABID | CCTGCCTATCTCTTTTGCATTCC | CACCTCCAAAAATACGCTGCC |
| OTUB1 | AGAGCGACCACATCCACATC | ATCGTAGTGTCCAGGCCGGT |
| OTUB2 | GAGCGGTCGGGTGTATTCTC | CTCATAGTGACCAGAGCCGC |
| OTUD1 | TCCACATCATTCCAGACGGC | TGGGCAGCAGCGATGATAAA |
| OTUD4 | GCTTTGCGTCTGAAAGGTGC | CATGGCGAGACTGAGAGTGC |
| OTUD5 | AGTTCAGCCTCGTCACCTG | CCCCTGCCGAGAACTGACTG |
| OTUD6B | GGAGGAAGCAACTCACCGAA | GCAGCTTTCTTTTCCCGTCTC |
| OTUD7A | TGATCCCCCTGACGGATTCT | TCAGCTTGGCTTCTAGCGAC |
| YOD1 | GCCATTTTGGAGTCCACCCG | GGACAGCCCCTGCAAAACA |
| BRCC3 | CGGGTACTCTACACTTGCTTCC | CCTCAATGGAGATGTGCTGGCT |
| STAMBP | GCTCAACCAGGTGGTAACCG | GACGGGGTGGAATGTCTTCA |
| STAMBPL1 | GGGACCATCGCAGTGACAAT | CCGACAGATGGAGCTTTGCT |
| MYSM1 | AGCCCAGATGGCTCTTATCG | CATGGGGACGCTATGGGAGAG |
