# Supplementary material for: Hispidulin Enhances TRAIL-Mediated Apoptosis via CaMKKβ/AMPK/USP51 Axis-Mediated Bim Stabilization
Source: Cancers (Basel). 2019 Dec 6;11(12):1960. doi: 10.3390/cancers11121960 (PMC6966507; doi:10.3390/cancers11121960)
Supplement: Supplementary file 1 [file cancers-11-01960-s001.zip › Supplementary data.pdf]

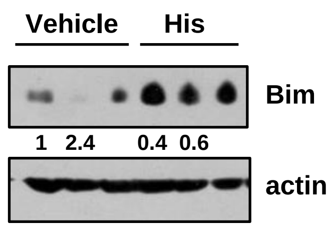

Supplementary Figure 1. Effect of hispidulin on Bim expression in tumor tissues. Protein expression was measured by western blotting.

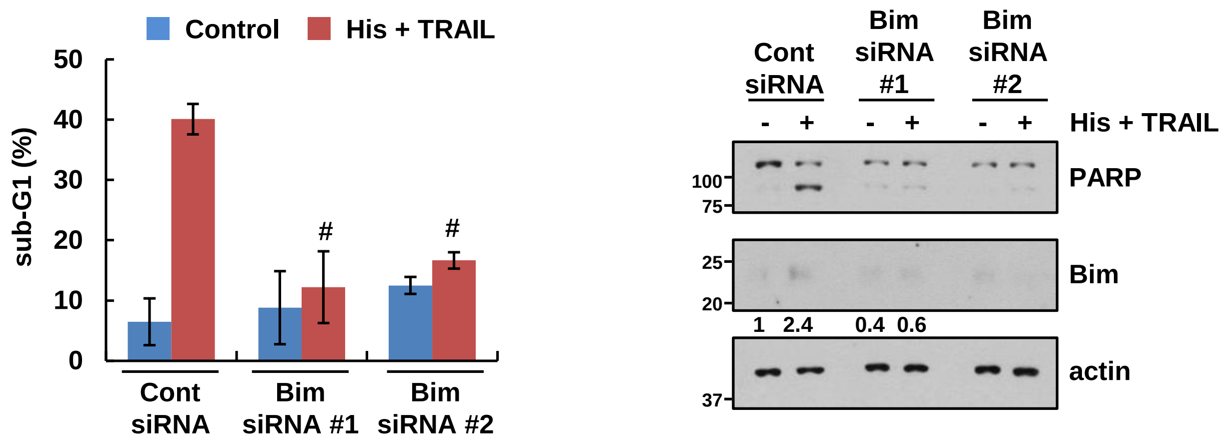

Supplementary Figure 2. Bim is critical for hispidulin plus TRAIL-induced apoptosis. A498 cells were transiently transfected with Bim siRNA or control siRNA. After transfection, cells were exposed to 30 μM hispidulin and 50 ng/ml TRAIL for 24 h. The level of apoptosis was analyzed by measuring the sub-G1 fraction by flow cytometry. The protein levels were determined by Western blotting. The values represent the mean ± SD from three independent experiments. # $p < 0.05$ compared to the hispidulin plus TRAIL in control siRNA.

Figure 1A

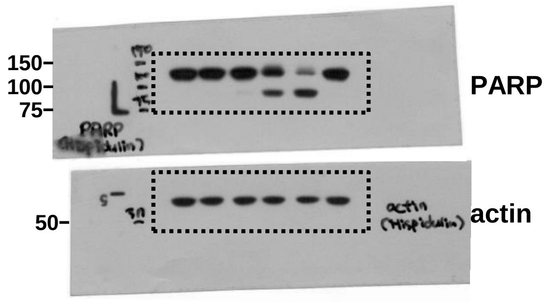

Figure 1B

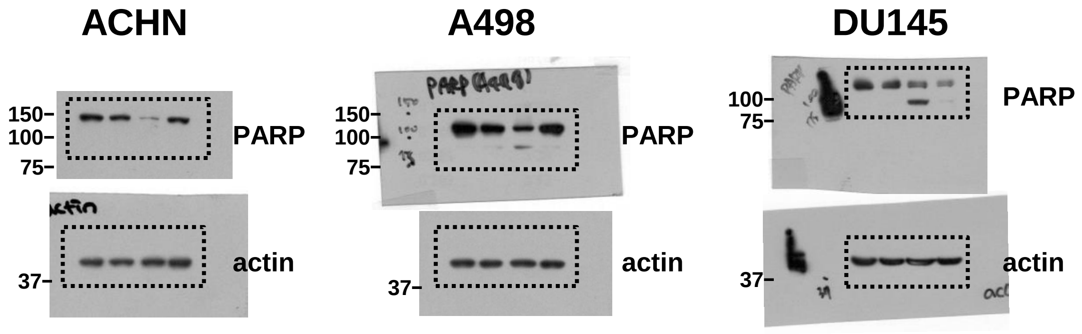

Figure 1F

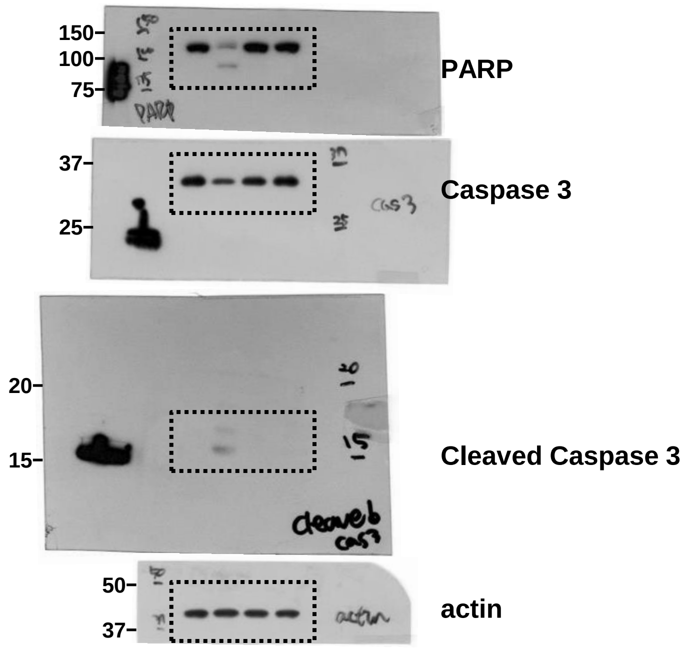

Supplementary Figure 3. Uncropped image for Figure 1.

Figure 3B

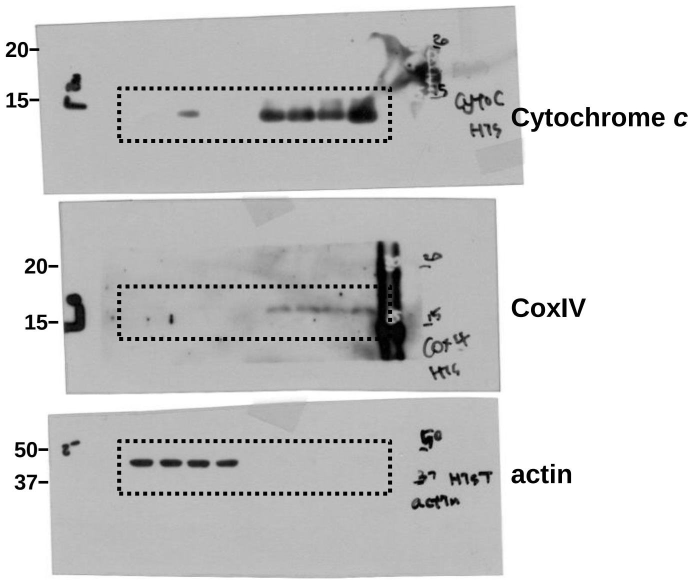

Figure 3C

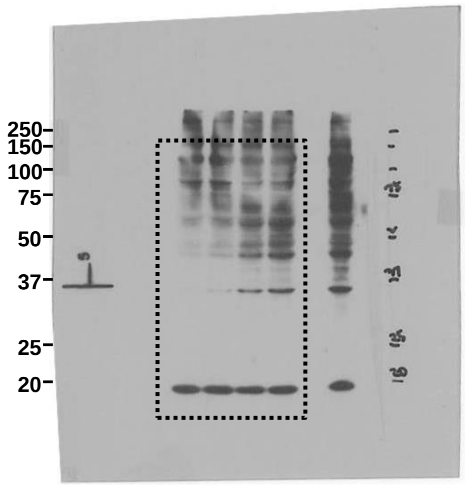

Figure 3D

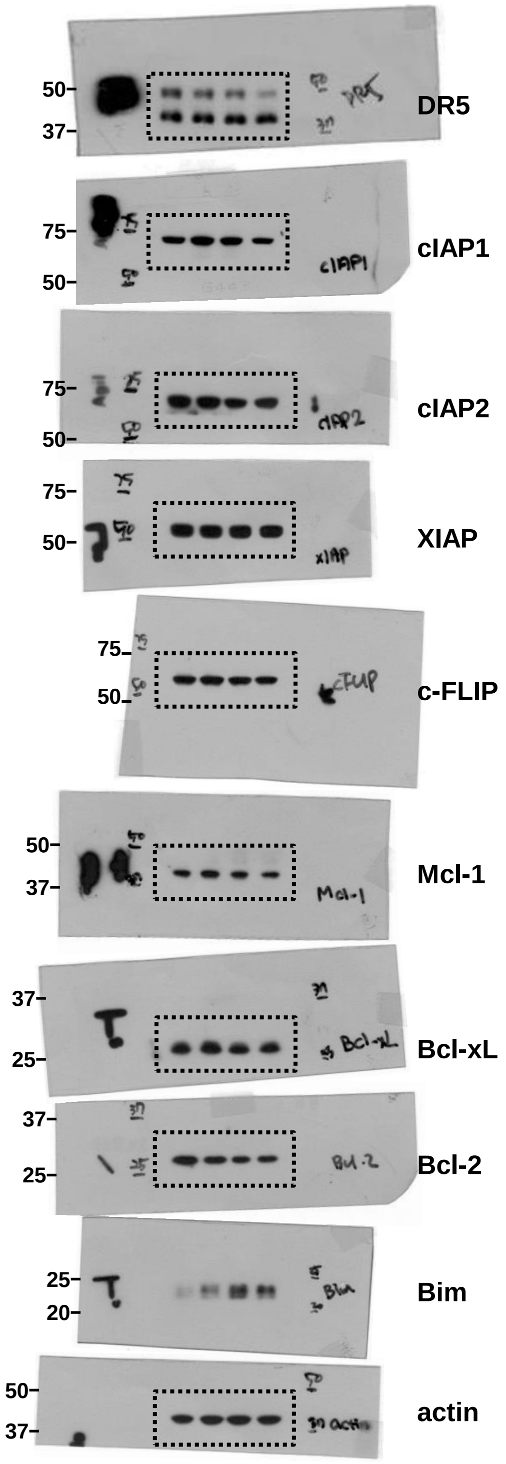

Figure 3E

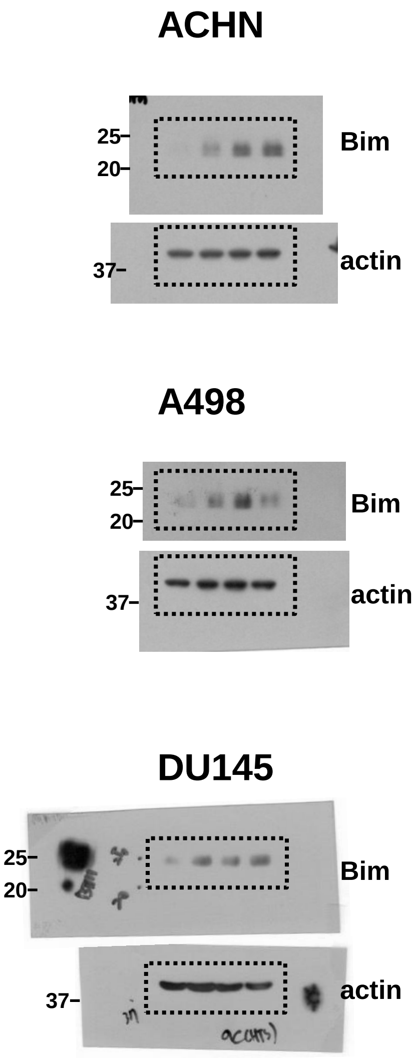

Supplementary Figure 4. Uncropped image for Figure 3.

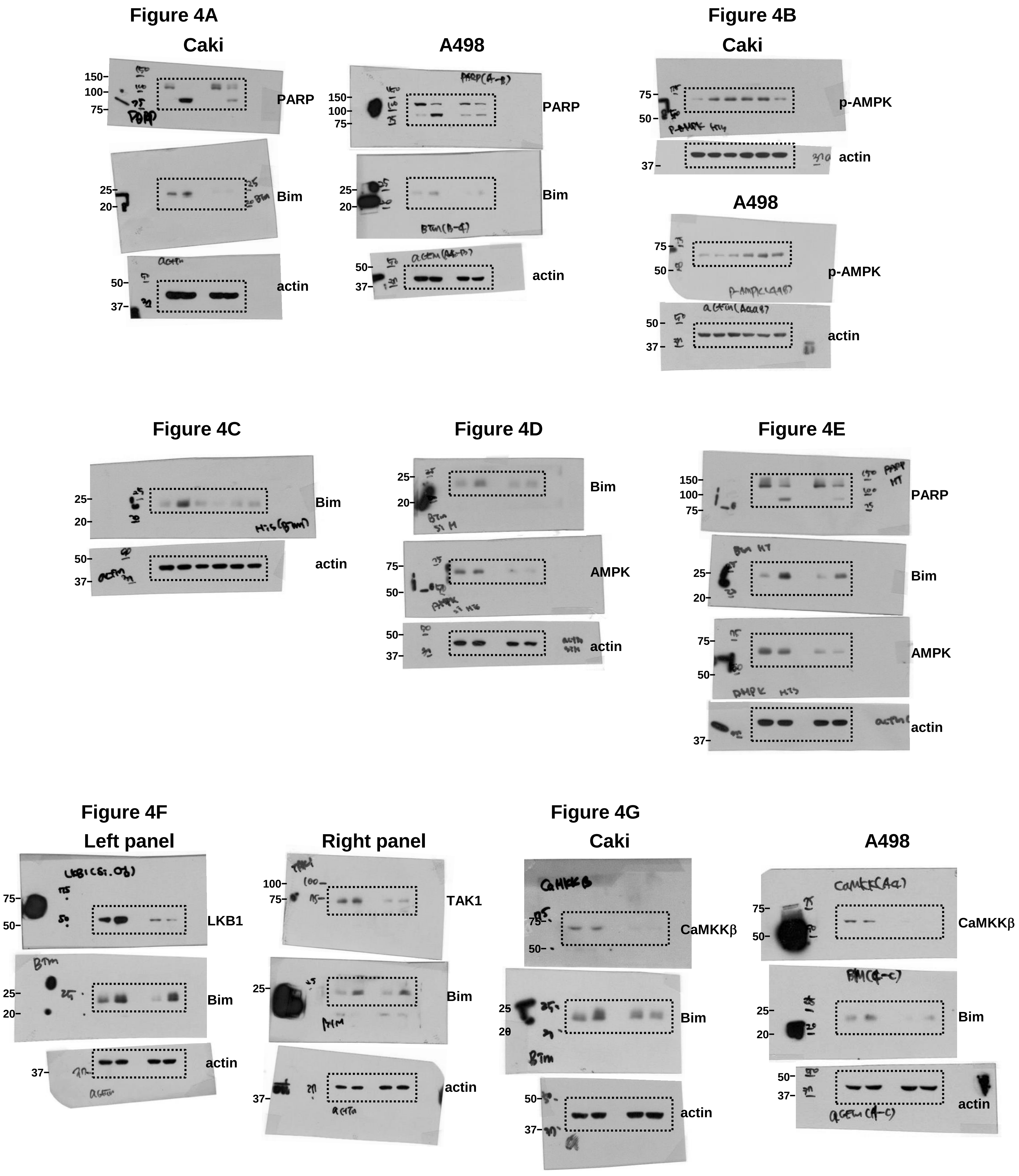

Supplementary Figure 5. Uncropped image for Figure 4.

Figure 5A

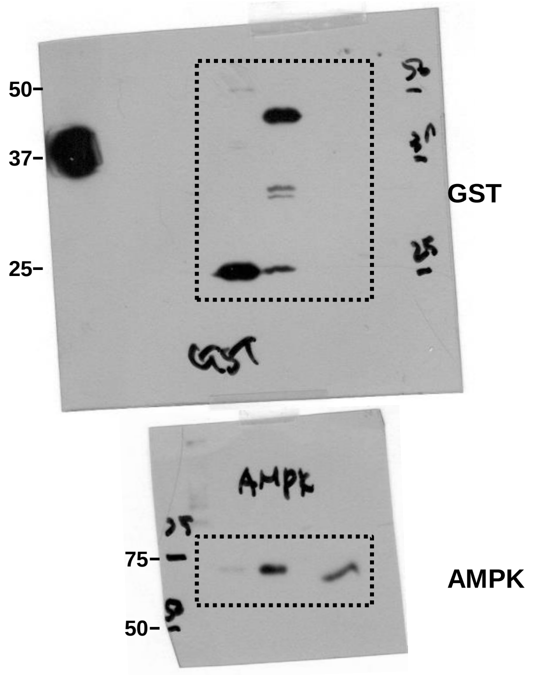

Figure 5B

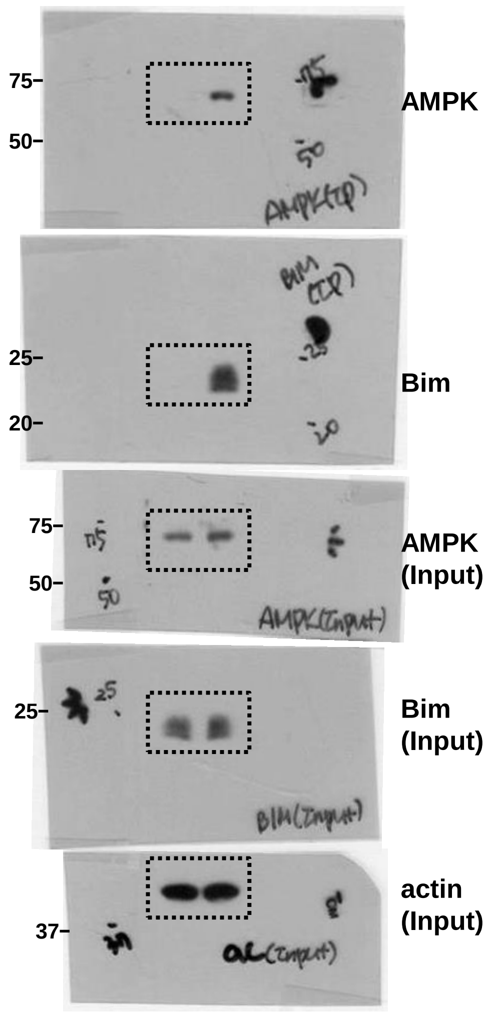

Figure 5C

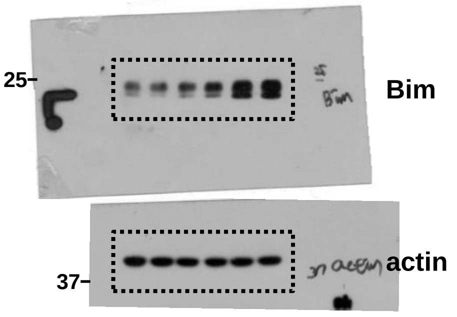

Figure 5D

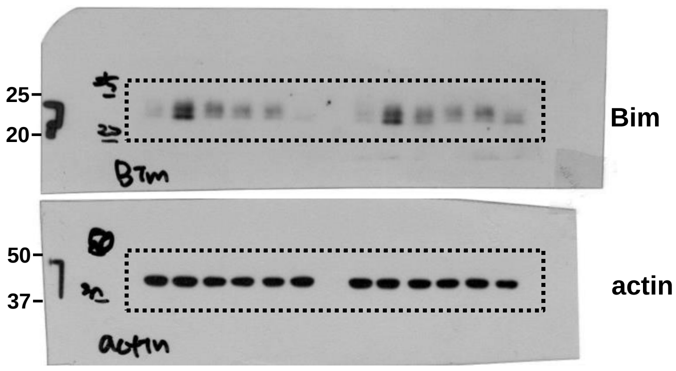

Figure 5E

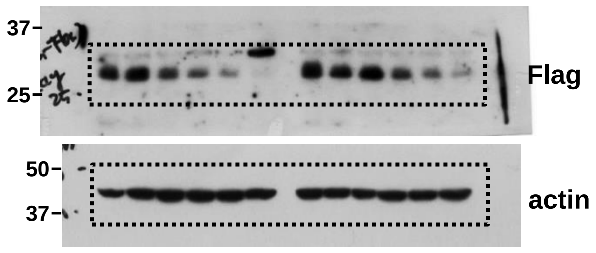

Figure 5F

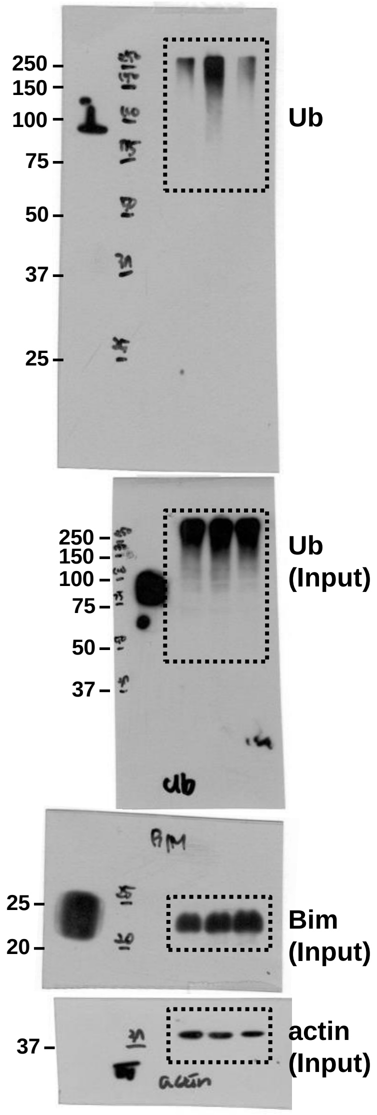

Figure 5G

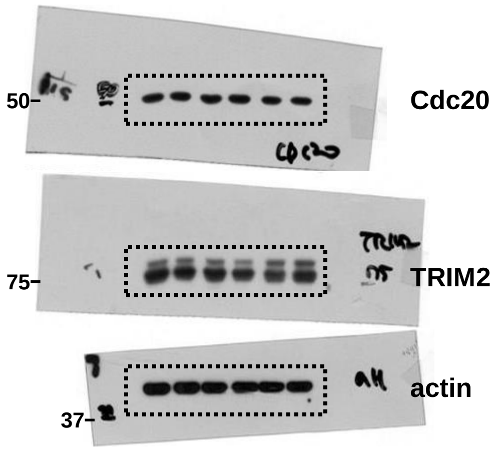

Supplementary Figure 6. Uncropped image for Figure 5.

Figure 6B

Left panel

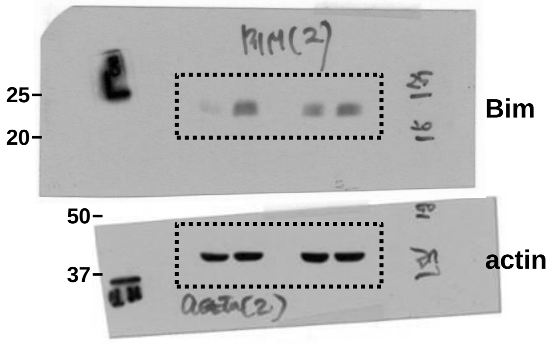

Right panel

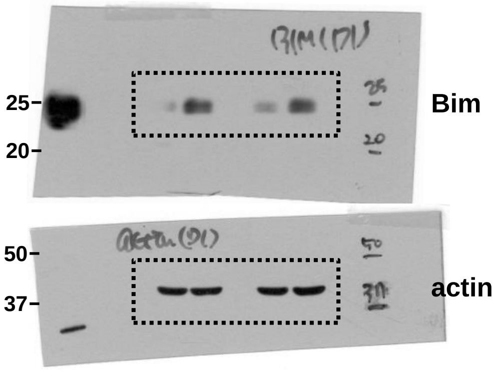

Figure 6C

Caki

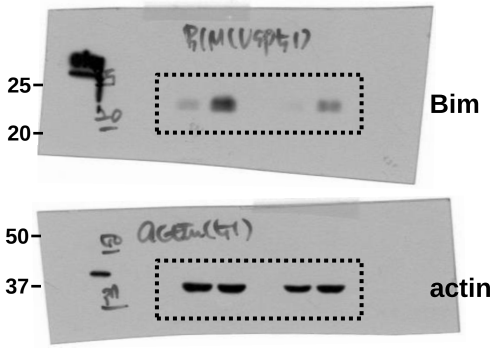

A498

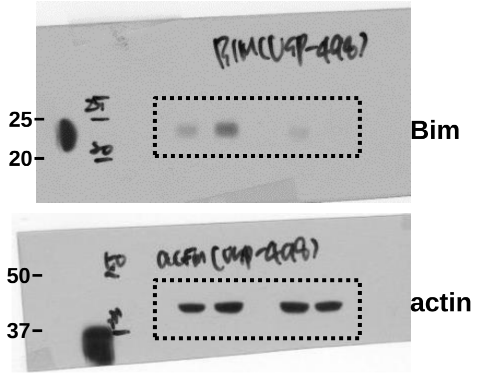

DU145

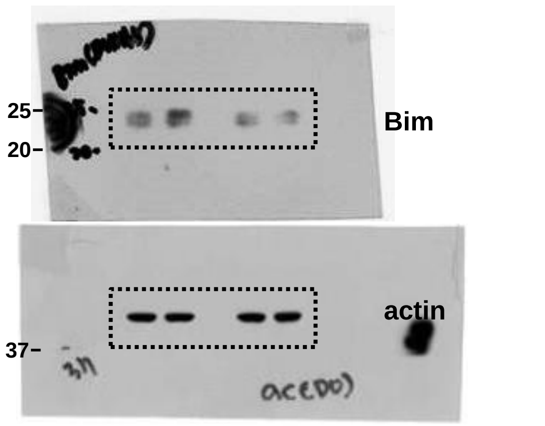

Figure 6D

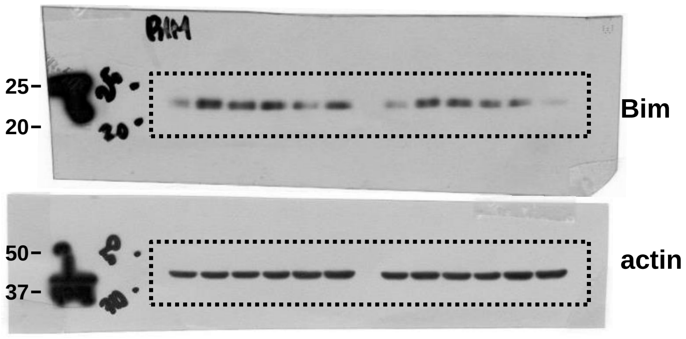

Figure 6F

Caki

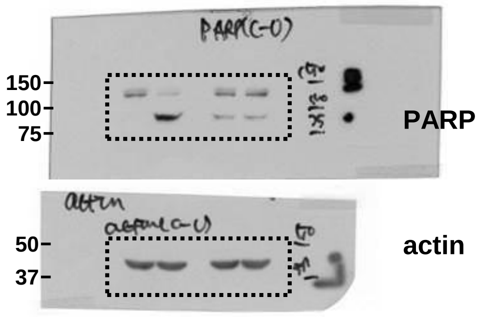

A498

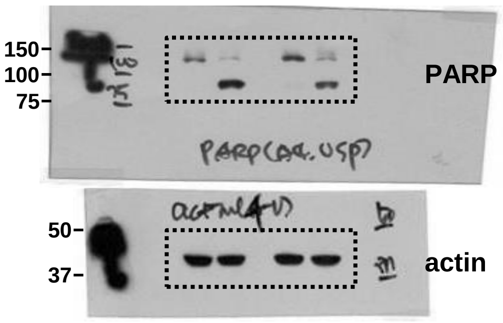

DU145

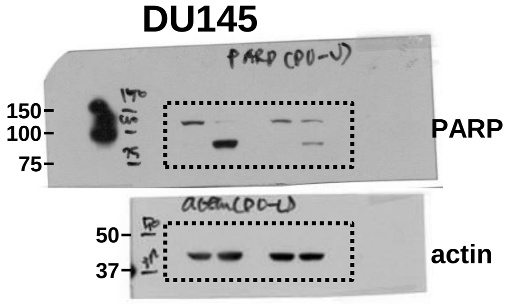

Supplementary Figure 7. Uncropped image for Figure 6.

Supplementary Figure 1.

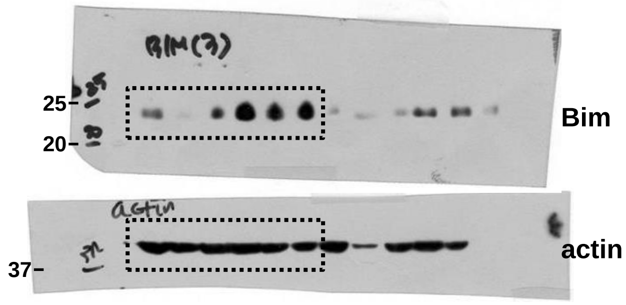

Supplementary Figure 2.

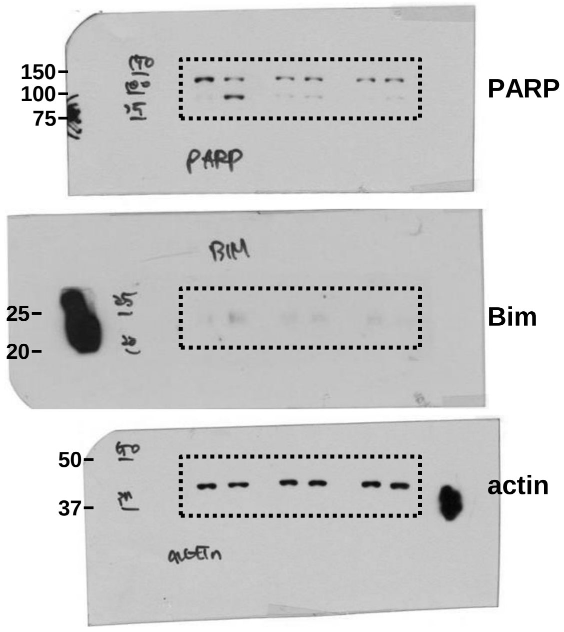

Supplementary Figure 8. Uncropped image for supplementary Figure 1 and 2.
